# Health Literacy, Socioeconomic Status and Vaccination Uptake: A Study on Influenza Vaccination in a Population-Based Sample

**DOI:** 10.3390/ijerph19116925

**Published:** 2022-06-06

**Authors:** Patrizio Zanobini, Chiara Lorini, Saverio Caini, Vieri Lastrucci, Maria Masocco, Valentina Minardi, Valentina Possenti, Giovanna Mereu, Rossella Cecconi, Guglielmo Bonaccorsi

**Affiliations:** 1 Department of Health Sciences, University of Florence, 50134 Florence, Italy; chiara.lorini@unifi.it (C.L.); guglielmo.bonaccorsi@unifi.it (G.B.); 2 Cancer Risk Factors and Lifestyle Epidemiology Unit, Institute For Cancer Research, Prevention and Clincal Network (ISPRO), 50139 Florence, Italy; s.caini@ispro.toscana.it; 3 Epidemiology Unit, Meyer Children’s University Hospital, 50139 Florence, Italy; vieri.lastrucci@unifi.it; 4 Centro Nazionale per la Prevenzione delle Malattie e la Promozione della Salute, Istituto Superiore di Sanità, 00161 Rome, Italy; maria.masocco@iss.it (M.M.); valentina.minardi@iss.it (V.M.); valentina.possenti@iss.it (V.P.); 5 Department of Prevention, Azienda Unità Sanitaria Locale (AUSL) Toscana Centro, 50122 Florence, Italy; giovanna.mereu@uslcentro.toscana.it (G.M.); rossella.cecconi@uslcentro.toscana.it (R.C.)

**Keywords:** health literacy, influenza vaccination, socio-economic determinants, high-risk groups, HLS-EU-Q6

## Abstract

Background: Health Literacy (HL) has been recently hypothesized to affect the relationship between socio-economic status (SES) and health conditions. However, to date no study has yet assessed the potential contribution of HL in the pathway through which SES affects influenza vaccination status. We aim to examine the relationships among HL, SES factors, and influenza vaccination uptake in Tuscan (Italy) residents belonging to different high-risk groups (HRGs) for influenza. Methods: The study was performed within the Tuscan population sample selected in the Italian Behavioral Risk Factor Surveillance System in 2017–2018. HL was assessed using the Italian version of the 6-items European Health Literacy Survey Questionnaire (HLS-EU-Q6). Mediation analyses were conducted using SES variables as independent variables, influenza vaccination status as dependent variable and HL as mediator variable. Results: A total of 3278 people belonged to HRGs for influenza. In the whole sample, 19.4% of the participants were vaccinated against influenza. Participants who were not employed or had a poor financial status were more likely to be vaccinated against influenza (OR 1.56, 95%CI 1.26–1.94, *p* < 0.001, and OR 1.21, 95%CI 1.00–1.48, *p* = 0.047 respectively). HL did not mediate the relationship of any of the independent variables with influenza vaccination status. Conclusions: Some SES determinants resulted to influence influenza vaccination uptake, while HL did not affect the likelihood of influenza vaccination uptake among HRGs. Universal health care systems, as in the case of Italy, offering influenza vaccination free of charge to HRGs help in reducing inequalities and mitigating HL demands.

## 1. Introduction

Influenza is a highly contagious respiratory disease caused by influenza viruses. Hospitalization and death occur most frequently among high-risk groups (HRGs) like older people and those with underlying chronic health conditions [1,2]. To reduce the number of flu-related complications and deaths, influenza vaccination is annually recommended to everybody aged ≥ 6 months who do not have contraindications [3]. 

In the attempt of increasing vaccination coverage and investigating the determinants of vaccine acceptance and/or vaccine hesitancy, many factors must be taken into account. Among them, socioeconomic status (SES) and health literacy (HL) appear to be two potential strong candidates. 

Robust evidence from many studies supports an association between low SES and different health conditions and behaviors [4,5,6], although the pathway by which SES influences health has not been clarified yet [7]. The specific role of SES as predictor of vaccine acceptance is still debated: in fact, while some empirical studies report that vaccine hesitancy is more frequent among people with a high SES, other researchers report the opposite or observe no relation, especially regarding educational level [8]. 

HL is defined as “the combination of personal competencies and situational resources needed for people to access, understand, appraise and use information and services to make decisions about health. It includes the capacity to communicate, assert and act upon these decisions” [9]. The contribution of one’s HL on vaccine acceptance is still unclear. In fact, HL acts as the balance between individual, community and population skills and the system’s complexity: people’s abilities are mediated by environmental demands and situational complexities, i.e., the context in which HL is developed and applied [10,11], and HL is therefore a key determinant of health and wellbeing. Limited HL is associated with unhealthy behaviors, worse health outcomes, a lower use of preventive health services and an increased use of medication, fewer healthy choices, and impaired ability to manage long-term conditions [9,12,13]. 

In this perspective, HL has been recently hypothesized to affect the relationship between SES and health status: HL might partially mediate the effect of SES on some health outcomes, health-related behaviors, and on the access and use of healthcare [14,15,16]. As for the relationship between SES and vaccination status, to date no study has assessed, however, the potential contribution of HL in the pathway through which socio-economic determinants affect influenza vaccination status. 

In this study, we aimed to help filling this knowledge gap and examined the relationships among HL, SES factors (education level, occupation, economic status, and citizenship), and influenza vaccination uptake in Tuscan (Italy) residents belonging to different high-risk groups for influenza. In particular, we sought to investigate whether HL level is a predictor of influenza vaccination uptake and a mediator of the possible association between SES factors and influenza vaccination uptake.

## 2. Materials and Methods

### 2.1. Study Population, Sampling Criteria and Data Collection

The study has been performed within the Tuscan population sample selected in the Italian Behavioral Risk Factor Surveillance System (PASSI—Progress by local health units toward a healthier Italy), an ongoing nationwide surveillance system. Since 2008, PASSI collects data on the prevalence of some major behavioral risk factors for non-communicable chronic diseases and the adherence to important preventive measures in the Italian adult population (18–69 years old). It is jointly carried out by the Italian Local Health Units (LHUs) and regions, coordinated by the Italian National Institute of Health (ISS). In each LHU, a random sample is drawn monthly from the enrolment list of residents, stratified by sex and age proportionally to the size of the respective stratum in the general population. The LHU data are merged and analyzed to obtain regional and national representative estimates. Further details on the PASSI surveillance systems have been described elsewhere [17,18,19]. The eligible population is selected among residents aged 18 to 69 years enrolled on the LHU lists who have a phone number and available to be interviewed by telephone. Data were collected using the CATI (computerized assisted telephone interview) technique. The exclusion criteria are the inability to speak and understand Italian, inability to be interviewed (e.g., severe cognitive impairment, and severe disabilities), and hospitalization or institutionalization during the survey period.

In brief, a letter explaining the purpose of the surveillance system is sent to the homes of the selected persons. Telephone numbers are obtained from the enrollment list, telephone directories, the GPs, or the respondents themselves when they call to make an appointment after receiving the letter. At least 6 attempts are made to call on different days of the week (including weekends) and at different times of the day; if a person cannot be reached, a substitute of the same sex and age group is randomly extracted.

Telephone interviews are held by trained personnel from the public health departments of each LHU using a standardized questionnaire. Informed consent was obtained from all the participants. The data were anonymized and electronically recorded in a national database. Interviews recorded during each year were collected in annual dataset. 

The core questionnaire comprises 114 questions grouped in 12 modules. Additional modules can be introduced to answer to specific health questions proposed by regions or other institutions (Ministry, University, etc.). In Tuscany, an additional module aimed at investigating HL was in fact administered in 2017 and 2018, proposed by the HL research group of the University of Florence.

The present study considered the data referred to 7157 persons, that is the regional representative sample of Tuscan residents in 2017–2018, which is described elsewhere [20]. For the purpose of this study only data from residents at high-risk of influenza (3278, 46% of the whole sample) were analysed. High-risk groups (HRG) were identified according to the seasonal national and regional recommendations:

- adults aged 65 years and older; for the aim of the research, the age class considered was therefore 65–69 (the upper limit for age of the PASSI sample), defined in this article as “young seniors”;
- individuals with chronic medical conditions (ICMC): persons who suffer from chronic pulmonary (including asthma) or cardiovascular diseases, renal or hepatic diseases, or diabetes, or cancer, and persons with body mass index (BMI) greater than 30 kg/m^2^;
- at-risk professional categories (RPC): healthcare workers, military personnel, police, firefighters, schools’ personnel, breeders, veterinarians, slaughterers.
- For all the above described HRGs, in Italy the influenza vaccination is guaranteed free of charge by the national healthcare system [21].

### 2.2. Measurements

Socio-demographic characteristics included in this analysis were sex, age (which, depending on the type of analysis performed, was modelled as a continuous variable or grouped into 18–34, 35–49, or 50–69 years age bands), education level (dichotomized as: “low” for secondary school diploma or lower; “high” for high school diploma or higher). Furthermore, financial status was assessed asking the respondents whether they experienced difficulties in getting at the end of the month with the personal/ familial financial resources available (“With the financial resources at your disposal (from your own or family income) how do you get to the end of the month?, with four possible answers, then grouped into two categories for analytical purposes: “very easily”, “quite easily” (coded together as “good”), “with some difficulties”, or “with many difficulties” (coded as “poor”). For occupational status, two categories were identified: employed vs. not employed. Lastly, the Nationality (Italian or foreign) was considered. 

HL level was assessed using the Italian version of the 6-items European Health Literacy Survey Questionnaire (HLS-EU-Q6) [22,23], which is the short-short form of the 47-items tool (HLS-EU-Q47). It is a self-reported tool with Likert-type responses (“very easy”, “fairly easy”, “fairly difficult”, “very difficult”) and a final score that can be used to measure HL in general populations. For each item, the following scores were considered: “very easy” = 4; “fairly easy” = 3; “fairly difficult” = 2; “very difficult” = 1. “Don’t know” or refusal were recoded as missing. The scale final score was the mean value and varied between 1 and 4. Only respondents who answered at least 5 items were considered. According to the final score (x), three possible levels of HL have been defined: inadequate HL (1 ≤ x ≤ 2); problematic HL (2 < x < 3); sufficient HL (3 ≤ x ≤ 4). In this study, HL was treated as a dichotomous variable grouping together problematic and sufficient HL levels (sufficient/problematic HL).

Influenza vaccination status was assessed by asking respondents if they had been vaccinated in the previous epidemic seasons. 

### 2.3. Statistical Analysis

**Figure 1:**
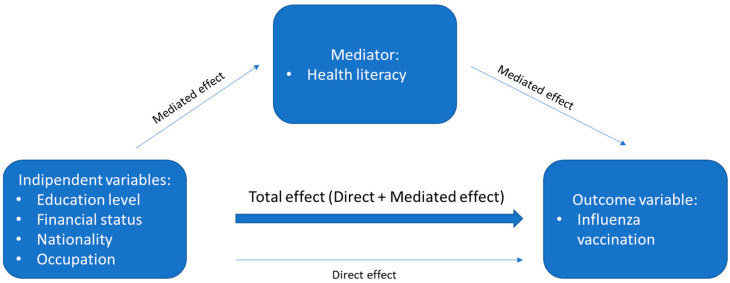
Hypothesized causal pathway of HL as a mediator of the association between socio-economic status and influenza vaccination uptake.

Data was presented as percentage or as mean, standard deviation and median. The causal pathways, which consider SES variables, HL and influenza vaccination were specified a priori and formally tested by means of mediation analysis. In particular, the causal pathways were aimed at assessing whether HL mediates the association between each pair of variables, represented by an exposure of interest (the following SES variables were individually tested: education level, occupational status, financial status, and nationality) and the outcome (influenza vaccination status) in HRGs (Figure 1). The analysis proceeded in two steps. First, two logistic regression models were separately fitted to investigate the association between the exposure and HL (“mediator model”), and between the exposure and the outcome (“outcome model”, which also includes the mediator as covariate). All these models were adjusted for age (entered as continuous variable), sex and for each of the SES variables. Second, the outputs of the mediator and outcome models were used to feed a mediation model fitted by using the “mediate” command (package “mediation”) in the R software (version 4.0.0) [24]. The mediate function computes the total effect of the exposure on the outcome and decomposes it into an indirect effect (average causal mediation effect—ACME) and a direct effect (average direct effect—ADE). In terms of interpretation, the indirect effect reflects the magnitude of the effect that is transmitted through the mediator, whereas the direct effect accounts for all the other possible causal chains. In addition, the “mediate” command yields the “proportion mediated”, which should be interpreted as an estimate of the percentage of the total effect that is exerted through the mediator. For each analysis, an α level below 0.05 was considered as significant.

## 3. Results

A total of 3278 people from the Tuscan sample interviewed in the PASSI surveillance belonged to HRGs for influenza. In particular, 1351 participants were aged 65–69, 2476 were ICMC, and 544 belonged to RPC. Sixty-seven point eight percent belonged to only one HRG, 31.0% to two HRGs and 1.2% to all the three HRGs. A descriptive analysis of the collected data is presented in Table 1. In the whole sample, 19.4% of the participants was vaccinated against influenza in the previous year. Influenza vaccination uptake was higher among young seniors (28.9%) and lower among RPC (11.8%).

In terms of socio-economic characteristics, 39.0% was not employed, 38.3% had low education level, 44.0% reported a poor financial status, and 5.2% had a foreign citizenship. About 8.0% of participants had an inadequate HL level (see Table 1).

### 3.1. Association between Health Literacy and SES Factors (Independent Variables) (Mediator Models)

The results of the mediator models for education, occupational status, financial status and nationality considering the total sample and the different HRGs are reported in Table 2. Participants belonging to a RPC were only considered in the total sample analysis (Table 2) because none of those with an inadequate level of HL was vaccinated and therefore a logistic regression analysis was not feasible. 

As for the mediator model, education level, occupation, financial status, and foreign nationality were associated with HL level in the total sample (Table 2). In particular, participants with a low education level had an increased odds ratio (OR) of having inadequate HL compared with those with a high education level (OR 2.14, 95% CI 1.61–2.85, *p* = <0.001). As for the relationship between financial status and HL, participants having a poor financial status had an increased likelihood of having inadequate HL compared with those with good financial status (OR 1.94 95% CI 1.46–2.59, *p* < 0.001). As for nationality, having a foreign nationality was significantly associated with HL level in the total sample (OR 3.41 95% CI 2.21–5.28, *p* < 0.001). Similar results for low education level, having poor financial status and having a foreign nationality were observed in the mediator models carried out in young seniors and in ICMC (see Table 2).

As for occupation status, being unemployed was significantly associated with inadequate HL level in the total sample (OR 1.42 95% CI 1.05–1.93, *p* = 0.023) and in the ICMC (OR 1.54 95% CI 1.12–2.11, *p* = 0.008) but no significant association was observed in the young senior sample.

### 3.2. Association between Influenza Vaccination Status, Health Literacy and SES Factors (Outcome Models and Mediation Analysis)

No significant association was observed between HL and participants’ vaccination status either in the total sample (*p* > 0.05) and in all the subsamples considered (see Table 3). 

As far as the considered independent variables were concerned, occupational status was significantly associated with influenza vaccination status in the total sample and in the ICMC (see Table 3). Particularly, unemployed participants were more likely to be vaccinated against influenza (OR 1.56, 95%CI 1.26–1.94, *p* < 0.001 in the total sample, and OR 1.59, 95%CI 1.25–2.01, *p* < 0.001 in the ICMC subsample). 

Having a poor financial status was positively associated with being vaccinated in the total sample (OR 1.21, 95%CI 1.00–1.48, *p* = 0.047)

Education level and foreign nationality were not significantly associated with influenza vaccination status. 

All the mediation models showed no significant mediation role played by HL in the relationship between independent variables and influenza vaccination status in the total sample and in all the subsamples considered (see Table 4).

## 4. Discussion

The identification of individual predictors of influenza vaccination uptake represents a challenge for the public healthcare systems, in terms of considering them in projecting and realizing interventions aimed at increasing people’s adherence to vaccination. Among them, SES and HL are of great interest because they are related with equity and inequalities in health and—just in case of HL and of some SES predictors—are modifiable. Therefore, this study tried to explore in depth the relationships among HL, SES and influenza vaccination uptake in Tuscan (Italy) residents that belonged to different high-risk groups (HRGs) for influenza. In the investigated sample, vaccination uptake was about 18%, with higher acceptance among young seniors (26.4%) and lower among RPC (15.8%). According to our results: i. HL was not significantly associated with influenza vaccination uptake; ii. Occupational and financial status were significantly associated with vaccination uptake; iii. SES was significantly associated with HL, in all its components; iv. HL was not a mediator of the relationship between SES and influenza vaccination uptake. 

Concerning the role of SES and HL in predicting influenza vaccination uptake, scientific literature reported contrasted results, depending on the country in which the studies have been conducted and on the target groups. In particular, some Authors have described a positive association between SES and vaccination coverage (the lower the SES, the lower the influenza vaccination uptake) while others have found no association [25,26,27,28]. Contrasting results have been described for the relationship of HL and vaccination coverage too, possibly because of the inconsistency of the tools used to measure HL [29,30]. 

Our results showed associations only between the financial and employment status and the vaccination uptakes in HRGs

As suggested by Lucyk et al. [26], in countries with universal, publicly-funded health care systems, as is the case of Italy, the burden exerted by SES on influenza vaccination uptake is little or absent, since it is reduced by an equitable access, free of charge, for all the categories provided by law. 

Regarding employment, we found a significant association between been unemployed and vaccination uptake: not employed people showed a higher likelihood of getting vaccinated. This result could be explained considering that employed people may lack enough time to access the vaccination services despite the intention to get the vaccine, as described by other studies [30]. Another potential explanation could be related to the differences in clinical health conditions, that is the fact that ICMC could be contemporarily affected by a lower capacity to work and by a greater need to be vaccinated, owing to their health conditions. We also found a weak association between financial status and vaccination uptake. People with a poor financial status presented a higher likelihood of getting vaccinated than those with a good one. Probably, this aspect is related with the previous one: that is, people with worse financial situation more likely have a temporary and less remunerated job, thus having more time available to get the vaccine. 

Information about vaccines tend to be complex. Functional health literacy (both prose literacy and numeracy skills), as well as critical and evaluation skills, are required to seek out the right information, especially considering fake news reported on the Internet and on social media [31,32]. 

In our study HL was not associated with vaccination status; a possible explanation of this results is the fact that vaccination is actively offered to HRGs by the General Practitioners (GPs) and primary care services, thus mitigating the role of HL in the vaccination uptake decision. Another possible explanation for this lack of association between HL and vaccination is that the skills that people need to navigate, understand, appraise and apply vaccine-related information in order to make decision regarding vaccination uptake could be dissimilar to those needed for other health issues. In this sense, the specific assessment of vaccine literacy instead of general HL could better contribute to analyse the predictors of vaccination uptake or hesitancy. In fact, vaccine literacy has been defined as “not simply knowledge about vaccines, but also developing a system with decreased complexity to communicate and offer vaccines as sine qua non of a functioning health system” [33]. To date, vaccine literacy has been rarely investigated and very few are the tools specifically devoted to measure it, although the COVID-19 pandemic has brought out the relevance of this issue and fostered many new studies. The development of an ad hoc vaccine literacy measurement tool could be useful to understand whether specific functional, critical and interactive competencies in the field of vaccination could influence vaccine confidence and the consequent uptake. [29,30,34,35,36,37,38]. 

This work has some strengths and limitations. Among the former, the use of a large and representative sample of the Tuscan population and the assessment of multiple dimensions of SES. Regarding the limitations, firstly, some HRGs have not been considered in our analysis (i.e., caregivers) since the PASSI questionnaire did not contain any specific item that could identify them. Moreover, none of the respondents in the sample of RPC with an inadequate HL level reported to be vaccinated, so that we could not include the RPC sample in the mediation analysis. Another limitation is that the PASSI surveillance did not take into account the population over 69 years old, so limiting the subgroup of older to younger seniors (65–69 years old) as well as the generalisability of the results to the older population groups. 

Another important issue is that we could not measure the confidence towards influenza vaccine in the residents’ GPs: in fact, GPs’ demographic and professional characteristics could explain differences in vaccination coverage of their assisted people only partially, while other factors can account most [39]: some of them are known to be skeptic or even contrary towards vaccination in general or some particular types of vaccines [40]. Vaccine-hesitant physicians are likely to less recommend vaccines to their patients [41,42].

Moreover, our study reported only one HL subjective measure (HLS-EU-Q6), although McCormack et al. [43] and Lorini et al. [44] suggested to include multiple tools in the study design for more in-depth assessment of HL.

Lastly, we considered HL the mediator of the relationship between SES and vaccine uptake based on previous studies [14,15]. However, for future research the hypothesis that SES could be the mediator variable between HL and vaccine uptake should be investigated. 

## 5. Conclusions

In conclusion, in our study some SES determinants resulted to influence influenza vaccination uptake, while HL did not affect the likelihood of influenza vaccination uptake among HRGs. Universal health care systems offering influenza vaccination free of charge to HRGs help in reducing inequalities as well as in mitigating HL demands. However, vaccination coverage in HRGs remains too low and interventions are needed to reach the minimum level of 75% established by the ECDC [45]. In future studies, it could be useful to investigate the role of other potential predictors—both at individual, such as vaccine literacy, as well as at contextual level, such as HL and vaccine confidence of the persons’ social network—to better understand how and how much they can influence vaccination uptake, especially among HRGs.

## Figures and Tables

**Table 1:** Descriptive analysis of collected variables -total sample, young seniors, individuals with chronic medical conditions (ICMC) and at-risk professional categories (RPC)- in the whole sample and by influenza vaccination status.

Variables	TOTAL		YOUNG SENIORS (65–69 Years Old)		ICMC		RPC	
	% * N = 3278	Vaccinated (%) ° = 19.39	% * N = 1351	Vaccinated (%) ° = 28.86	% * N = 2476	Vaccinated (%) ° = 21.10	% * N = 544	Vaccinated (%) ° = 11.76
Age								
18–34	8.65	7.97 [5.32–11.76]	-		8.76	8.60 [5.46–13.31]	14.11	5.22 [2.09–12.44]
35–49	24.87	11.36 [9.38–13.68]	-		25.70	13.08 [10.69–15.90]	42.78	6.78 [4.27–10.61]
50–69	66.48	23.88 [22.09–25.77]	100	28.86 [26.47–31.37]	65.54	25.92 [23.79–28.17]	43.11	18.84 [14.13–24.67]
Sex								
Male	47.27	20.09 [18.12–22.21]	48.69	30.04 [26.56–33.78]	49.57	21.28 [19.03–23.71]	33.22	15.93 [11.06–22.42]
Female	52.73	18.76 [17.01–20.65]	51.31	27.73 [24.53–31.18]	50.43	20.93 [18.77–23.26]	66.78	9.69 [7.12–13.05]
Education level								
High	61.68	17.58 [15.96–19.32]	49.36	28.39 [25.02–32.02]	58.65	19.30 [17.32–21.44]	92.75	11.73 [9.21–14.84]
Low	38.32	22.20 [19.93–24.66]	50.64	29.07 [25.72–32.66]	41.35	23.55 [20.98–26.34]	7.25	12.14 [5.03–26.49]
Occupation								
Employed	60.99	14.12 [12.64–15.73]	33.44	22.26 [18.59–26.42]	59.65	15.34 [13.56–17.31]	100	11.76 [9.31–14.76]
Not employed	39.01	27.66 [25.22–30.24]	66.56	32.16 [29.13–35.34]	40.35	29.66 [26.84–32.64]	-	-
Financial Status								
Good	56.02	18.54 [16.77–20.44]	58.59	27.31 [24.03–30.47]	51.02	20.10 [17.91–22.49]	71.4	13.74 [10.64–17.58]
Poor	43.98	20.75 [18.7–22.97]	41.41	31.70 [27.93–35.73]	48.98	22.31 [20.01–24.79]	28.6	7.18 [3.97–12.65]
Nationality								
Italian	94.75	19.45 [18.08–20.89]	97.06	28.66 [26.24–31.20]	94.11	21.28 [19.65–23.01]	96.72	11.99 [9.46–15.09]
Foreign	5.25	18.27 [12.91–25.21]	2.94	30.04 [17.42–46.65]	5.89	28.61 [12.78–26.29]	3.28	7.61 [1.07–38.52]
HL level								
Sufficient/Problematic	92.04	18.91 [17.54–20.35]	89.58	28.34 [25.83–30.98]	91.26	20.51 [18.88–22.24]	97.03	12.12 [9.59–15.20]
Inadequate	7.96	24.98 [19.92–30.84]	10.42	33.34 [25.79–41.84]	8.74	27.3 [21.56–33.90]	2.97	0

* column percentage; ° row percentage, [] 95% confidence interval.

**Table 2:** Mediator Models: association (logistic regression analysis adjusted for age, sex and SES factors) between Health Literacy (dependent variable: inadequate Vs sufficient/problematic) and SES factors (education, occupation, financial status, nationality; independent variables) in high-risk groups (total sample, young seniors, and individuals with chronic medical conditions). HRG: high risk group; ICMC: individuals with chronic medical conditions; OR: Odds Ratio; CI: Confidence Interval. SES: socioeconomic status.

Independent Variables	HRG: Total				Young Seniors				ICMC			
Education	OR	95%CI		*p*	OR	95%CI		*p*	OR	95%CI		*p*
High	1	-	-	-	1	-	-	-	1	-	-	-
Low	2.14	1.61	2.85	<0.001	2.64	1.71	4.07	<0.001	1.84	1.35	2.49	<0.001
Occupation												
Employed	1	-	-	-	1	-	-	-	1	-	-	-
Unemployed	1.42	1.05	1.93	0.023	1.14	0.69	1.87	0.616	1.54	1.12	2.11	0.008
Financial Status												
Good	1	-	-	-	1	-	-	-	1	-	-	-
Poor	1.94	1.46	2.59	<0.001	1.69	1.14	2.51	0.009	2.10	1.52	2.90	<0.001
Nationality												
Italian	1	-	-	-	1	-	-	-	1	-	-	-
Foreign	3.41	2.21	5.28	<0.001	2.47	1.08	5.66	0.32	3.33	2.07	5.35	<0.001

**Table 3:** Outcome Models: association (logistic regression analysis adjusted for age and sex) between influenza vaccination status (vaccinated vs. not vaccinated; dependent variable), SES factors (education, occupation, financial status, nationality) and Health Literacy (independent variables) in high-risk groups (total, young seniors, and individuals with chronic medical conditions). HRG: high risk group; ICMC: individuals with chronic medical conditions; OR: Odds Ratio; CI: Confidence Interval. SES: socioeconomic status.

Independent Variables	HRG: Total				Young Seniors				ICMC			
Education	OR	95%CI		*p*	OR	95%CI		*p*	OR	95%CI		*p*
High	1	-	-	-	1	-	-	-	1	-	-	-
Low	0.92	0.71	1.19	0.522	0.87	0.67	1.14	0.319	0.87	0.70	1.09	0.224
Occupation												
Employed	1	-	-	-	1	-	-	-	1	-	-	-
Unemployed	1.56	1.26	1.94	<0.001	1.21	0.88	1.66	0.238	1.59	1.25	2.01	<0.001
Financial Status												
Good	1	-	-	-	1	-	-	-	1	-	-	-
Poor	1.21	1.00	1.48	0.047	1.28	0.98	1.67	0.066	1.21	0.97	1.50	0.088
Nationality												
Italian	1	-	-	-	1	-	-	-	1	-	-	-
Foreign	1.21	0.77	1.90	0.403	1.27	0.60	2.82	0.551	1.13	0.69	1.87	0.623
HL												
Sufficient/problematic	1	-	-	-	1	-	-	-	1	-	-	-
Inadequate	1.09	0.79	1.51	0.587	1.11	0.74	1.66	0.605	1.12	0.79	1.61	0.499

**Table 4:** Mediation analyses: Mediation effect of health literacy in the association between SES factors (education, occupation, financial status, nationality; independent variables) and influenza vaccination status in high-risk groups (HRGs): Total, Young seniors, and Individuals with chronic medical conditions (ICMC).

	Education				Occupation				Financial Status				Nationality			
HRG:Total sample	estimate	95% CI		*p*	estimate	95% CI		*p*	estimate	95% CI		*p*	estimate	95% CI		*p*
Total Effect	−0.02	−0.06	0.01	0.20	0.05	0.03	0.07	<0.001	0.24	0.00	0.05	0.046	0.03	−0.05	0.08	0.44
ACME *	0.00	−0.01	0.00	0.59	0.00	0.00	0.00	0.67	0.00	0.00	0.00	0.62	0.00	0.00	0.00	0.60
ADE **	−0.02	−0.06	0.01	0.19	0.05	0.03	0.07	<0.001	0.02	0.00	0.05	0.050	0.02	−0.05	0.08	0.45
Young seniors	estimate	95% CI		*p*	estimate	95% CI		*p*	estimate	95% CI		*p*	estimate	95% CI		*p*
Total Effect	−0.03	−0.10	0.03	0.35	0.03	−0.03	0.08	0.26	0.04	0.00	0.08	0.07	0.04	−0.15	0.18	0.61
ACME *	0.00	0.00	0.00	0.65	0.00	0.00	0.00	0.95	0.00	0.00	0.00	0.65	0.00	0.00	0.01	0.65
ADE **	−0.02	−0.10	0.03	0.35	0.03	−0.03	0.08	0.27	0.04	−0.03	0.08	0.07	0.03	−0.15	0.17	0.62
ICMC	estimate	95% CI		*p*	estimate	95% CI		*p*	estimate	95% CI		*p*	estimate	95% CI		*p*
Total Effect	−0.02	−0.06	0.01	0.27	0.06	0.29	0.08	<0.001	0.03	−0.01	0.05	0.10	0.02	−0.07	0.09	0.64
ACME *	0.00	0.00	0.00	0.54	0.00	0.00	0.04	0.53	0.00	0.00	0.00	0.61	0.00	0.00	0.00	0.53
ADE **	−0.02	−0.06	0.01	0.26	0.06	0.02	0.07	<0.001	0.02	−0.01	0.05	0.11	0.02	−0.07	0.09	0.65

* ACME (average causal mediation effect) ** ADE (average direct effect).

## Data Availability

The data presented in this study are available on request from the corresponding author. The data are not publicly available due to privacy restrictions.
